# Tryptophan-supplemented diet modulates the metabolic response of European seabass (*Dicentrarchus labrax*) juveniles reared under space-confined conditions and submitted to acute inflammation

**DOI:** 10.1007/s10695-024-01427-1

**Published:** 2024-12-11

**Authors:** Diogo Peixoto, Juan Antonio Martos-Sitcha, Benjamín Costas, Rita Azeredo, Juan Miguel Mancera

**Affiliations:** 1 CIIMAR - Centro Interdisciplinar de Investigação Marinha E Ambiental, Av. General Norton de Matos S/N 4450-208, Matosinhos, Portugal; 2 ICBAS - Instituto de Ciências Biomédicas Abel Salazar, Universidade Do Porto, Porto, Portugal; 3 Departamento de Biología, Facultad de Ciencias del Mar y Ambientales, Instituto Universitario de Investigación Marina (INMAR), CEIMAR-Universidad de Cádiz, Cádiz, Spain

**Keywords:** Fish metabolism, Stress, Tryptophan nutrition, Space-confined conditions, Welfare, European seabass

## Abstract

The energetic costs of being in stressful conditions require the involvement of hormones associated with metabolic support, which may also influence immune function. The present work aimed to explore the links between tryptophan nutrition and metabolic responses in European seabass (*Dicentrarchus labrax*) held under space-confined conditions, and subsequently submitted to an immune challenge. To study that, two dietary treatments were evaluated, i.e. control diet (CTRL) and CTRL-based diet supplemented with tryptophan (0.3%; TRP) to fish under space-confinement conditions (10 kg/m^3^) or not (5 kg/m^3^). Dietary treatments were offered for 15 days after which fish were intraperitoneally injected (i.p.) with *Photobacterium damselae piscicida*. Liver was sampled before the immune challenge (at end of the nutritional trial, 0 h) and at 4, 24, 48 and 72 h post-injection. Fish-fed TRP exhibited distinct metabolic profiles compared to those fed CTRL diets, particularly in energy metabolism and stress response. CTRL-fed fish in space-confined conditions showed a gradual reduction of lipid oxidative enzyme activity post-injection, pointing to acute stress-induced lipid catabolism feedback. In contrast, stressed fish fed TRP under same rearing conditions presented reduced glucose levels and cortisol production but unchanged 3-hydroxiacil-CoA dehydrogenase (EC 1.1.1.35, HOAD) activity patterns, suggesting an inhibitory and modulatory role of tryptophan in stress response. Discriminant analysis revealed that fish fed TRP under space-confined conditions resembled fish CTRL-fed under non-stressful conditions. These findings suggest that tryptophan dietary supplementation for stressed fish modulates their metabolic responses and potentially mitigates the negative effects of rearing stressful conditions after subsequent acute stress induced by an immune challenge.

**Supplementary Information:**

The online version contains supplementary material available at 10.1007/s10695-024-01427-1.

## Introduction

Immunonutrition has been defined as modulation of basal immune defences or immune response by feed ingredients (Grimble 2009). Appropriate feed and feeding regimes provide optimum health by linking nutrition to immunity, together with other areas such as biochemistry, physiology, microbiology and pathology. In several livestock activities, including aquaculture, functional diets including immune-enhancing nutrients (such as pigments, nucleotides, pre- and probiotics and amino acids) present modulatory properties on the immune system (Emadi et al. 2011; Estensoro et al. 2016; Grimble 2009; Li et al. 2009). Amino acids appear to be good candidates to be supplemented in aquafeeds to improve health and survival as well as a complementary strategy to improve vaccination effectiveness (Costas et al. 2008). Tryptophan is an essential amino acid that can only be assimilated through diet since fish are not able to synthetize it endogenously (Conceição et al. 2012; Herrera et al. 2019). This amino acid triggers modulatory effects in the neuroendocrine system mostly given its role as the precursor of the neurotransmitter serotonin (5-hydroxytryptamine (5-HT)), thereby modifying stress and behavioural responses, as well as the antioxidant system (Conceição et al. 2012; Li et al. 2009; Schreck et al. 2016). Most of the ingested tryptophan is catabolized primarily in the liver via the kynurenine pathway (around 95%), being converted into various metabolites, including niacin (vitamin B_3_), pyruvate or acetyl-CoA, and subsequently used as a source of energy (Herrera et al. 2019, 2021; Yao et al. 2011). Degradation of tryptophan into kynurenine by immune cells yields a range of different compounds known to play a crucial role in the regulation of immune responses (Le Floc'h et al. 2011). Alternatively, tryptophan can be converted into 5-HT by tryptophan hydroxylase and the impact of this neurotransmitter on the hypothalamus–pituitary–interrenal (HPI) axis depends greatly on the neuroendocrine condition of fish (Azeredo et al. 2019). It can either stimulate or inhibit the axis, leading to an increase or decrease in cortisol production by the interrenal cells. Thus, tryptophan has been described as able to attenuate the stress response in fish (Herrera et al. 2020; Hoseini et al. 2019; Lepage et al. 2002; Martins et al. 2013; Tejpal et al. 2009) via modulation of serotonergic activity, though the underlying mechanisms remain unclear (Herrera et al. 2021; Le Floc'h and Seve 2007). Complementary analytical approaches to the hereby presented experiment were recently published (Machado et al. 2022; Peixoto et al. 2024) and highlighted the upregulation of immune-related genes, reversal of stress-induced T cell suppression, and inhibition of bacterial injection–induced cortisol production during seabass inflammatory response in fish feeding a diet supplement with tryptophan for 15 days under space-confined conditions. These studies revealed changes in gene expression patterns of several endocrine players at pituitary level (tryptophan hydroxylase (*tph1α*), serotonergic receptor (*htr2a*), and pro-opiomelanocortin a-like (*pomca*) and head-kidney (glucocorticoid receptor 1 (*gr1)*)) suggesting the involvement of HPI axis on the outcome. Their transversal downregulation seems to be associated to an inhibition of adrenocorticotropic hormone (ACTH) release and subsequent cortisol production.

Studies on several European aquaculture target species, such as rainbow trout (*Oncorhynchus mykiss*), Atlantic salmon (*Salmo salar*), European seabass (*Dicentrarchus labrax*), gilthead seabream (*Sparus aurata*) and Senegalese sole (*Solea senegalensis*), have been conducted for the assessment of dietary tryptophan (TRP) supplementation of aquafeeds under stressful conditions (Hoseini et al 2019; Machado et al. 2022; Oyarzún-Salazar et al. 2022; Salamanca et al. 2020a, 2020b). However, these studies do not consider the metabolism and its associated costs, narrowing their focus to immune responses and disease resistance. The physiological response of fish to stressors or pathogenic microorganisms affects the organism as a whole and implies intense cell proliferation as well as the production of a plethora of immune- and endocrine-related proteins (e.g. antibodies, complement factors, enzymes, hormones), being a very high energy–consuming processes (Schreck et al. 2016; Tort 2011).

The intricate cooperation between regulatory systems is of major importance to understand which physiological mechanisms are involved in tryptophan-mediated effects on metabolism and the stress response. Energetic costs of stressful conditions require activation of other hormonal pathways involved in metabolic support, which may also influence immune function and vice versa. So, the main aim of the present work is to explore metabolic indicators in European seabass fed tryptophan-supplemented diets under space-confined conditions (stressful rearing situation) and subsequently submitted to an acute inflammation induced by intraperitoneal injection of *Photobacterium damselae piscicida* (*Phdp*). The results will be discussed in relation to the energetic dynamics behind a tryptophan dietary intervention within this particular context.

## Materials and methods

### Diets composition

The experimental diets were formulated and manufactured by Sparos Lda. located in Olhão, Portugal. The control diet (CTRL) was specifically formulated to meet the indispensable AA profile recommended for European seabass, as outlined by Kaushik (1998). Additionally, a variant of the CTRL-based diet was developed by supplementing it with 0.3% l-TRP (dry matter), at the expenses of wheat meal. Ingredient composition and AA profile of experimental diets were described by Machado et al. (2022) and by Peixoto et al. (2024).

### *Phdp* inoculum preparation

The bacterial challenge was performed using *Photobacterium damselae piscicida* (*Phdp*) strain PP3, isolated from yellowtail (*Seriola quinqueradiata*; Japan) by Doctor Andrew C. Barnes (Marine Laboratory, Aberdeen, UK). The bacterial inoculum was prepared following the methodology described by Machado et al. (2022), to a final concentration of 5 × 10^7^ cfu mL^−1^ previously obtained with pre-challenge trials (lethal dose 40%).

### Experimental design

**Fig. 1:**
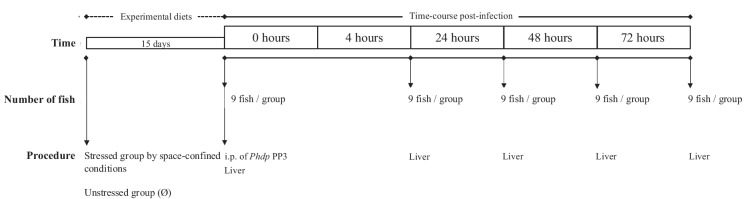
Experimental setup of the feeding trial under space-confined conditions followed by acute inflammation challenge induced by *Phdp* treatment

European seabass juveniles (12.02 ± 2.77 g) were randomly distributed in 16 (52 l) and maintained in two independent recirculating seawater systems (eight tanks each, 22 *per* tank; density of 5 kg m^3^) at the following environmental and constant conditions: temperature 20 ± 0.5 °C; salinity 32‰; photoperiod 10:14-h dark:light). In one system (eight tanks), fish served as control (Ø) for the density factor (5 kg/m^3^). In the other system, fish were kept under stressful conditions induced by space confinement (i.e. eight tanks with a density of 10 kg/m^3^ in 26 L and 13-cm height) by lowering the water level. Fish were fed the two dietary treatments previously described (Machado et al. 2022; Peixoto et al. 2024), twice a day, with a daily average ration of 2% of body weight for 15 days. At the end of this period, nine fish *per* treatment (undisturbed group; 0 h) were euthanized and liver samples were collected for the evaluation of hepatic metabolites levels and enzymatic activities (Fig. 1). The remaining fish (nine fish *per* treatment) were intraperitoneally injected with 100 µL of the *Phdp* inoculum (temperature 24 ± 0.5 °C; salinity 32; photoperiod 10:14-h dark:light) and similarly sampled at 4, 24, 48 and 72-h post-injection. This environmental temperature was selected in order to mimic temperatures observed during *Phdp* outbreaks in aquaculture farms. Fish were fasted 24 h before sampling or *Phdp* treatment. No mortality was observed during the trial. The experimental trial was directed by trained scientists (following FELASA category C recommendations) and conducted according to the guidelines on the protection of animals used for scientific purposes from the European directive 2010/63/UE.

### Hepatic samples preparation

To assess both metabolite levels and enzymatic activities, each liver sample was divided into two portions and then homogenized. One liver portion was weighed and homogenized by ultrasonic disruption with 7.5 volumes of ice-cold 0.6 N perchloric acid, neutralized using 1 M potassium bicarbonate and then centrifuged 30 min at 3220 × *g*, 4 °C, and supernatant was used to metabolite determination (Martínez-Antequera et al. 2023). The second portion of liver was weighed and homogenized by ultrasonic disruption with 10 volumes of ice-cold stopping buffer containing 50 mM imidazole–HCl (pH 8.5), 1 mM 2-mercaptoethanol, 50 mM sodium fluoride, 4 mM EDTA, 5 mM phenylmethanesulfonyl fluoride and 250 mmol L^−1^ sucrose (final pH 7.5). Afterward, samples were centrifuged for 30 min at 10,000 × *g*, 4 ^−^°C, and the supernatant was used in enzyme assays and protein analyses (Herrera et al. 2019).

### Hepatic metabolite determination

Liver lactate and triglyceride (TAG) levels were determined spectrophotometrically using commercial kits (Spinreact 1001311 and 1001330, respectively). Tissue glycogen concentration was assessed as described by Keppler and Decker (1974), where tissue homogenates are incubated for 2 h at 37 °C with and without amyloglucosidase (Sigma-Aldrich A7420) to break down glycogen molecules into glucose. The complete methodology for the determination of these metabolites in the liver is fully described in Basto et al. (2023). Total glucose in both incubations was determined with a commercial kit (Spinreact 1001200), and glycogen content was expressed as glucose equivalents after free glucose subtraction using the Keppler and Decker (1974) method. Metabolite levels were determined using a PowerWaveTM 340 microplate spectrophotometer (Bio-Tek Instruments, Winooski, VT, USA) using Kjunior™ data analysis software.

### Hepatic enzyme activities

Total protein concentration was quantified using the Pierce BCA Protein Assay Kit as described by Costas et al. (2014), with slight modifications on sample dilution (1/4 v/v).

The following enzymes activities were evaluated to assess changes in (i) carbohydrate metabolism (hexokinase (HK), EC 2.7.1.1; pyruvate kinase (PK), EC 2.7.1.40; and glycogen phosphorylase total and active (tGP and aGP), EC 2.4.1.1); (ii) lipid metabolism (3-hydroxiacil-CoA dehydrogenase (HOAD), EC 1.1.1.35); and (iii) lactate pathway (lactate dehydrogenase (LDH), EC 1.1.1.27).

HK (EC 2.7.1.1) was measured by preparing an incubation solution with 50 mM imidazole–HCl (pH 8.0), 5 mM magnesium chloride hexahydrate, 0.15 mM β-nicotinamide adenine dinucleotide hydrate and 1 mM adenosine 5′-triphosphate disodium salt hydrate. Plus, 5 mM d-( +)-glucose and 0.15 U *per* well glucose-6-phosphate dehydrogenase were added to each sample in a 96-well plate. The kinetic reaction was read at 340 nm with one read each 26 s (total 20 cycles) at 37 °C.

PK (EC 2.7.1.40) was assessed using an incubation solution with 50 mM imidazole–HCl (pH 7.4), 100 mM magnesium chloride, 100 mM potassium chloride, 10 mM magnesium chloride hexahydrate, 1 mM adenosine 5′-diphosphate bis(cyclohexylammonium) salt and 0.15 mM β-nicotinamide adenine dinucleotide. After that, 0.5 mM phospho(enol)pyruvic acid cyclohexylammonium salt, 6.195 U *per* well and 0.025 mM fructose-1,6-biphosphate were added to each sample in a 96-well plate. The kinetic reaction was measured at 340 nm with one read each 26 s (total 20 cycles) at 37 °C.

Glycogen phosphorylase (EC 2.4.1.1; total GP [tGP] and active GP [aGP]) was determined using an incubation solution composed by 50 mM phosphate buffer (potassium dihydrogen phosphate; pH 7.0), 27 mM magnesium sulfate, 1.3 mM ethylenediaminetetraacetic acid, 0.5 mM sodium salt hydrate and 2.5 mM adenosine 5′-monophosphate sodium salt. Plus, 5 mg mL^−1^ glycogen, 0.55 U *per* well phosphoglucomutase, 0.223 U *per* well glucose-6-phosphate dehydrogenase and 0.1 mM glucose 1,6–biphosphate were added to each sample in a 96-well plate. aGP activity was measured with 10 mM caffeine present, whereas tGP activities were estimated without caffeine. The kinetic reaction was measured at 340 nm with previous incubation of 60 s and then read each 26 s (total 20 cycles) at 37 °C.

HOAD (EC 1.1.1.35) activity was determined using an incubation solution composed of 50 mM imidazole–HCl (pH 7.4) and 0.16 mM β-Nicotinamide adenine dinucleotide. Adding 0.12 mM acetoacetyl coenzyme A sodium salt hydrate, the kinetic reaction was read at 340 nm with one read each 26 s (total 20 cycles) at 37 °C.

LDH (EC 1.1.1.27) activity was measured using an incubation solution 50 mM imidazole–HCl (pH 8.5) and 2.5 mM β-nicotinamide adenine dinucleotide hydrate. Adding 6.25 mM l-( +)-lactic acid, the kinetic reaction was read at 340 nm with one read each 26 s (total 20 cycles) at 37 °C.

Enzyme activities were determined using a PowerWave™ 340 microplate spectrophotometer (Bio-Tek Instruments, Winooski, VT, USA) using Kcjunior™ data analysis software. Reaction rates of enzymes were determined by changes in absorbance from the reduction of NAD(P)^+^ to NAD(P)H. The reactions were started by addition of 15 μL homogenate at a pre-established protein concentration, omitting the substrate in control wells (final volume 275–295 μL).

### Data analysis

All results are expressed as mean ± standard deviation (SD). Shapiro–Wilk test was used for normality of variances, as well as Pearson skewness coefficient. Differences were tested by a multivariate ANOVA, with time (relative to injection), dietary treatment (CTRL and TRP) and stress (crowding conditions or not) as main factors. The ANOVA was followed by a post hoc Tukey HSD test to identify significant differences amongst groups. Statistical analyses were carried out using IBM SPSS v27.0 Statistics with a significance level of 95% (*p* ≤ 0.05). To discriminate and classify the existing groups, a multivariate canonical discriminant analysis (DA) was performed on the dataset to evaluate linear combinations of the original variables that would best separate the groups (discriminant functions) using Addinsoft XLSTAT 2022 system software. Each discriminant function explains part of total variance of the dataset and is loaded by variables contributing the most to that variation. Discriminatory effectiveness was assessed by Wilk’s λ test, and the distance between group centroids was measured by squared Mahalanobis distance, and Fisher’s *F* statistic was applied to infer significance.

## Results

### Hepatic metabolites content changes

**Fig. 2:**
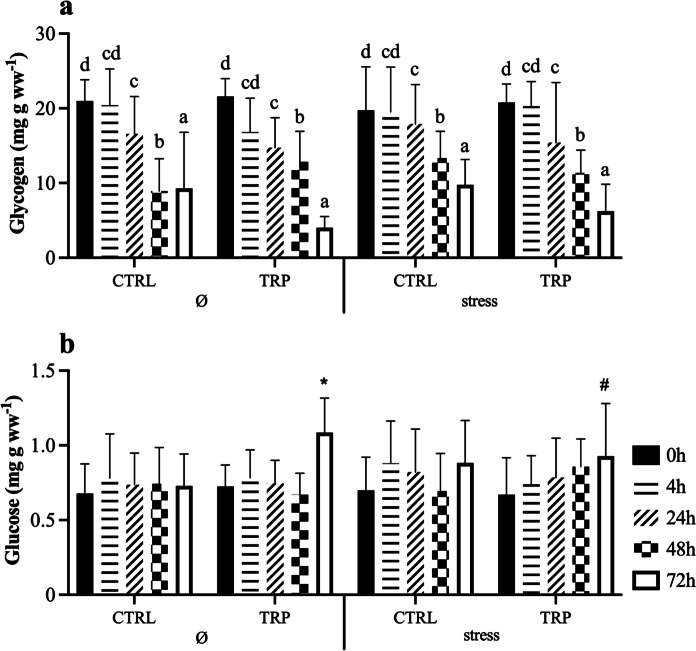
Hepatic glycogen (**a**) and glucose (**b**) contents of European seabass fed experimental diets under stressful conditions or not (Ø) or 15 days, and subsequently i.p. injected with *Phpd* and sampled before (0 h) and at 4, 24, 48 and 72 h post-injection. Values are presented as means ± SD (*n* = 9). *p*-values from three-way ANOVA (*p* ≤ 0.05). If an interaction was statistically significant, Tukey post hoc test was used to identify differences among treatments. Lowercase letters indicate differences attributed to sampling time. Different symbols denote significant differences between rearing conditions

**Fig. 3:**
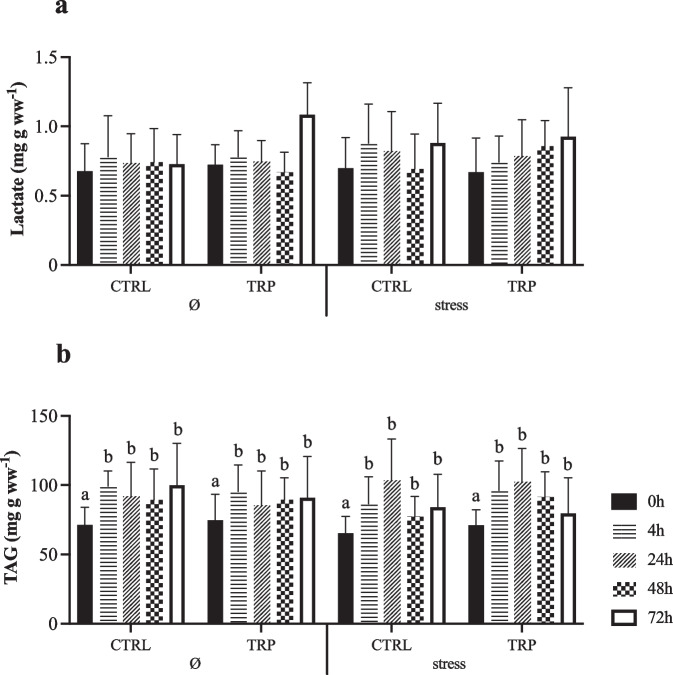
Hepatic lactate (**a**) and triglyceride (**b**) levels metabolites of European seabass fed experimental diets under stressful conditions or not (Ø) for 15 days, and subsequently i.p. injected with *Phpd* and sampled before (0 h) and at 4, 24, 48 and 72 h post-injection. Values are presented as means ± SD (*n* = 9). *p*-values from three-way ANOVA (*p* ≤ 0.05). If an interaction was statistically significant, Tukey post hoc test was used to identify differences among treatments. Lowercase letters indicate differences attributed to sampling time

The complete set of results is available in the Supplementary File (Table S1). Glycogen reserves decreased with injection time in all experimental groups (Fig. 2a). Stressful conditions were characterized by lower liver glucose content but only in fish fed tryptophan sampled 72 h post injection when compared to those unstressed (Fig. 2b). No significant differences were observed in hepatic lactate levels (Fig. 3a), whereas lipid-related metabolic parameters showed that triglyceride levels increased from 0 to 4 h in all experimental groups, maintaining the higher levels during the time post-injection evaluated (Fig. 3b).

### Hepatic metabolic-related enzymatic response

**Fig. 4:**
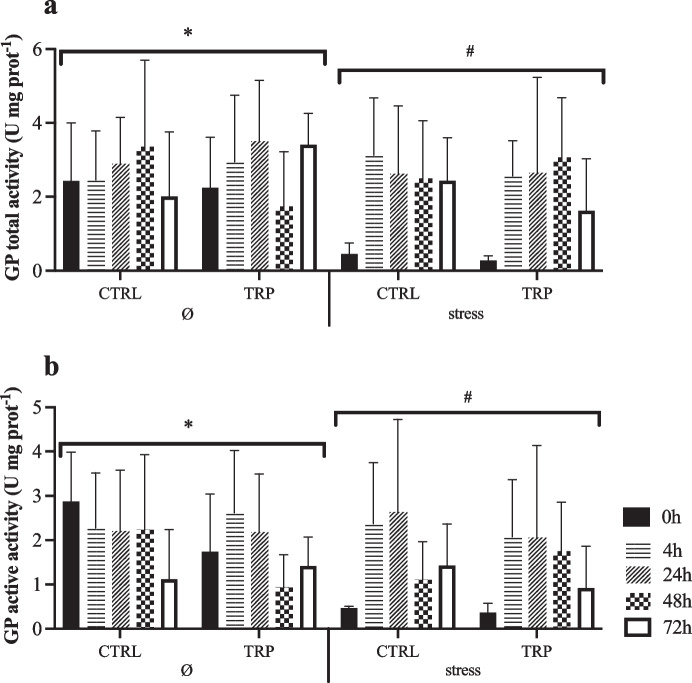
Hepatic metabolic-related glycogen phosphorylase (EC 2.4.1.1; **a** GP total and **b** GP active) activity of European seabass fed experimental diets under stressful conditions or not (Ø) for 15 days, and subsequently i.p. injected with *Phdp* and sampled before (0 h) and at 4, 24, 48 and 72 h post-injection). Values are presented as means ± SD (*n* = 9). *p*-values from three-way ANOVA (*p* ≤ 0.05). If an interaction was statistically significant, Tukey post hoc test was used to identify differences among treatments. Different symbols denote significant differences between rearing conditions

**Fig. 5:**
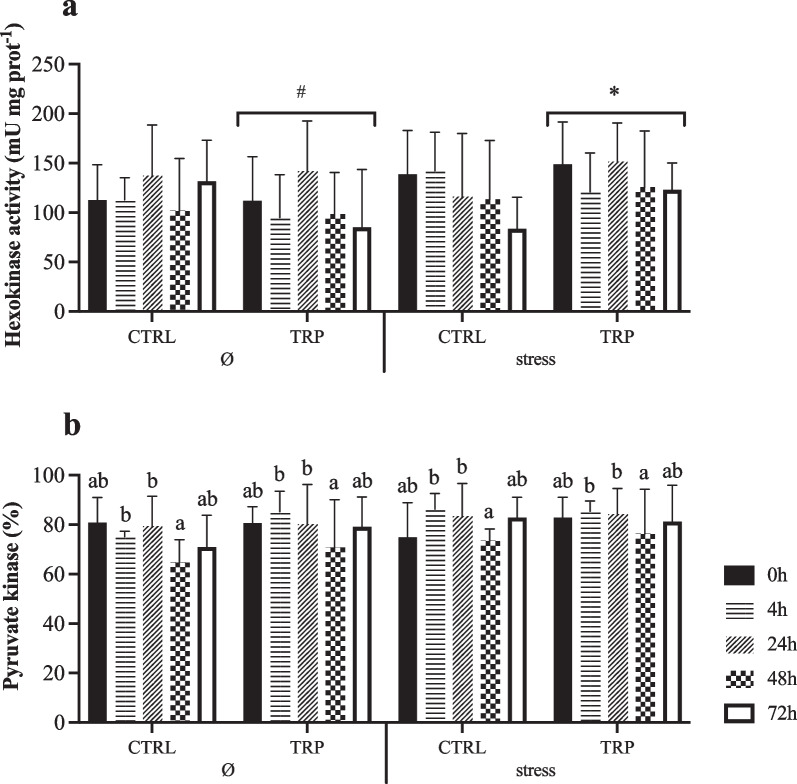
Hepatic metabolic-related enzymes activity of European seabass fed experimental diets under stressful conditions or not (Ø) for 15 days, and subsequently i.p. injected with *Phdp* and sampled before (0 h) and at 4, 24, 48 and 72 h post-injection. **a** Hexokinase (EC 2.7.1.1; HK) activity and (**b**) pyruvate kinase (EC 2.7.1.40; PK). Values are presented as means ± SD (*n* = 9). *p*-values from three-way ANOVA (*p* ≤ 0.05). If an interaction was statistically significant, Tukey post hoc test was used to identify differences among treatments. Lowercase letters indicate differences attributed to sampling time. Different symbols denote significant differences between rearing conditions

**Fig. 6:**
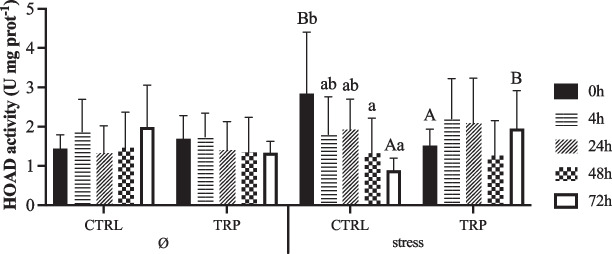
Hepatic metabolic-related 3-hidorxiacil-CoA dehydrogenase (EC 1.1.1.35; HOAD) activity of European seabass fed experimental diets under stressful conditions or not (Ø) for 15 days, and subsequently i.p. injected with *Phdp* and sampled before (0 h) and at 4, 24, 48 and 72 h post-injection). Values are presented as means ± SD (*n* = 9). *p*-values from three-way ANOVA (*p* ≤ 0.05). If an interaction was statistically significant, Tukey post hoc test was used to identify differences among treatments. Different capital letters stand for significant differences attributed to dietary treatment. Lowercase letters indicate differences attributed to sampling time

The complete set of results is available in the Supplementary File (Table S2). Hepatic GP (total and active) activities were both lower in stressed fish than in non-stressed fish in non-injected fish (0 h), regardless of other factors, with a clear trend of increase in this space-confined group after i.p. injection (Fig. 4a–b). HK activity enhanced in stressed fish fed TRP than in those unstressed, irrespective of sampling time (Fig. 5a). However, PK activity was lower at 48 h than at 4 and 24 h post-injection in all experimental conditions, returning to pre i.p. injection values after 72 h post-injection (Fig. 5b). Hepatic HOAD activity decreased from 0 to 72 h post-injection in stressed fish fed CTRL but remained stable in stressed fish fed tryptophan (Fig. 6). Moreover, the activity of this enzyme at 0 h was higher in stressed fish fed CTRL diet than in tryptophan counterparts, while the opposite occurred at 72 h post-injection.

### Overall correlation among experimental groups

**Fig. 7:**
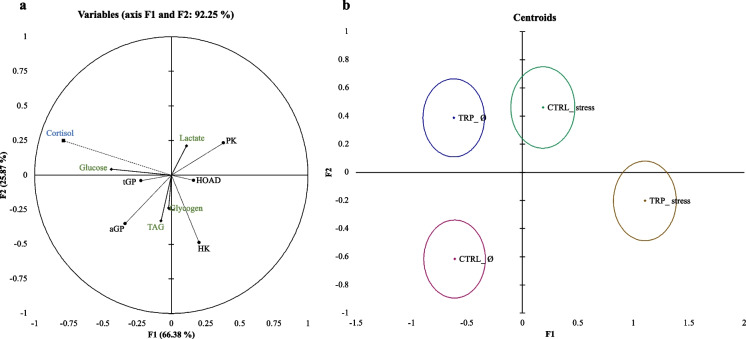
Canonical discriminant analysis of several parameters representative of the metabolic response of European seabass fed experimental diets for 15 days under control (Ø) or stressful conditions. **a** Correlation variables/factors (factor loads) for two main discriminant functions (F1 and F2). **b** Canonical discriminant scores of each group. Circle marks represent group centroids. (–-) Plasma cortisol levels, (^__^) hepatic metabolites values and (^**…**^) hepatic-related enzymatic activities. tGP, total glycogen phosphorylase (EC 2.4.1.1; tGP) activity; aGP, active glycogen phosphorylase (EC 2.4.1.1; aGP) acivity; TAG, triglycerides; HK, hexokinase (EC 2.7.1.1; HK) activity; HOAD, 3-hidorxiacil-CoA dehydrogenase (EC 1.1.1.35; HOAD) activity; and PK, pyruvate kinase (EC 2.7.1.40; PK) activity

The complete set of results is available in the Supplementary File (Table S3). A canonical discriminant analysis was performed to better understand stress- and inflammatory-associated energetic costs in European seabass provided with a dietary supplemented with TRP (Fig. 7). To enrich the analysis, plasma cortisol levels (from samples of the present experiment, but previously published by Machado et al. 2022) were added to the set of parameters. The overall performance of the analysis was characterized by a satisfactory discriminatory ability (Wilks λ = 0.518, *p* < 0.0001) with the first two discriminant functions accounting for 92.25% of the total dataset variability (Fig. 7a; F1 66.38% and F2 25.87%). When plotting the canonical discriminant scores of each group, a clear separation amongst them was observed (Fig. 7b), and the Mahalanobis distances between all pairs of groups were statistically significant (Table S3.2 from the Supplementary File). The shortest distance was that between unstressed fish fed tryptophan (TRP_Ø group) and stressed fish fed CTRL (CTRL_stress), which were positively loaded by plasma cortisol levels and negatively loaded by triglycerides values as well as HK and aGP activities (Table S3). Contrariwise, contrasting outcomes emerged for TRP_stress and CTRL_Ø which were positively loaded by aGP activity and negatively loaded by plasma cortisol and glucose levels as well as PK activity.

## Discussion

The physiological response of fish to stressors or pathogenic microorganisms involves metabolic mechanisms for energy consumption, wherein kynurenine metabolites, such as pyruvate and acetyl-CoA, are later used in Krebs cycle for energy production (Yao et al. 2011). One critical aspect of neuroendocrine or immune activation and the development of a stress response is the inherent high energy demand that compromises other primordial biological processes (Schreck et al. 2016; Tort 2011), since energy requirement to overcome stress response will be withdrawal from other physiological pathways, as growth performance, osmoregulation or reproduction. It is therefore of utmost importance to investigate energy substrates and the activity of enzymes when evaluating the efficacy of a dietary intervention that is expected to modulate both neuroendocrine and immune responses.

The liver is described as the main organ involved in intermediary metabolism in fish (Vijayan et al. 2010). In the present study, most metabolic changes observed were induced by i.p. injection of the bacterial inoculum. Given samplings’ timing corresponding to short periods post-injection (maximum of 72 h post-challenge), these changes are most likely reflections of an acute stress response, more than a result of immune stimulation.

Glycogen phosphorylase (GP) activity observed in this study was a clear response to the acute stress triggered by i.p. injection. In fish reared under space-confinement conditions, GP activity suddenly rose from stress-induced low levels to levels similar to those found in unstressed fish. Hepatic GP enzyme plays a central role in the maintenance of plasma glucose homeostasis, controlling the breakdown of glycogen to glucose 1-phosphate (Moon et al. 1999). Chronic stress (14 days of crowding conditions) in fish is known to suppress GP activity (Sangiao-Alvarellos et al. 2005; Tort 2011). In agreement with this, our results showed that GP activity (total and active) in fish under space-confinement conditions decreased respect to control fish. This fact could be the cause or the consequence of an adjustment of, among others, carbohydrate metabolism after a clear reduction of feed intake under this situation, although clear species-specific and age-specific dependence on the growth outcomes has been reported under different stocking conditions (De las Heras et al. 2015; Millán-Cubillo et al. 2016; Martos-Sitcha et al. 2019). It is an interesting remark that even after a chronic stress (15 days of crowding conditions) that suppressed this enzyme’s activity, the acute stress induced by *Phdp* treatment was able to reactivate glycogen breakdown into glucose. This is in agreement with other studies that have reported higher activities of glycolytic enzymes after an acute stressor exposure in fish (Mommsen et al. 1999; Iwama et al. 2006).

A significant rise post-injection in hepatic TAG levels was transversal to all experimental groups, regardless of dietary treatment or rearing conditions. Interestingly, marine fish primarily use lipids, such as triglycerides and cholesterol, as their principal and rapid energy source, as they poorly digest carbohydrates (Halver and Hardy 2020). It has been demonstrated that stressed seabream enhanced significantly hepatic TAG levels (Jerez-Cepa et al. 2019). These increases can be related with the role of stress hormones in stimulating hepatic TAG production through activation of certain enzymes involved in fatty acid synthesis, consequently increasing triglyceride synthesis and accumulation in the liver (Van Der Boon et al. 1991). Accordingly, in our study, the activity of the catabolic enzyme HOAD decreased as inflammation developed in stressed fish fed CTRL. This enzyme is used as a quantitative index for lipid catabolism (Driedzic 1992; Jayasundara et al. 2015) and a key regulatory enzyme of fatty acids beta oxidation (involved on the third step) for energy production (Turner et al. 2007). As suggested by decreasing glycogen levels, a seemingly glucose mobilization happened post-injection. Hence, an increase in glucose levels in liver was to be expected. The fact that hepatic glucose pool was observed to be unchanged might be related to its rapid conveyance to the circulatory system (Vijayan et al. 2010).

In the present study, no major metabolic changes associated with experimental dietary treatments were observed in fish reared at low stocking density after 15 days of feeding. Indeed, the few changes observed in the intermediary metabolism were those between dietary treatments in stressed fish as well as between unstressed and stressed fish fed TRP. Interestingly, TRP-fed fish exhibited lower HOAD activity before injection than those fed CTRL, but at 72 h post injection it was comparatively high. The gradual decreased activity observed in CTRL-fed fish sustains the fact that fish prefer lipid over carbohydrate sources, while handling stressful conditions, such as those associated to an i.p. bacterial injection that required a rapid energy source. Contrary, fish fed tryptophan showed a somewhat stable HOAD activity throughout the time-course trial. As mentioned before, parallel analytical approaches from Machado et al. (2022) and Peixoto et al. (2024) unveiled modulatory effects of tryptophan on cortisol levels of stressed fish that resemble those of unstressed fish. The maintenance of HOAD activity over the time course might be another modulatory role of tryptophan in stressed fish pointing to a homeostatic condition.

Canonical discriminant analysis provides clear evidence that under space-confined conditions, fish fed the CTRL and TRP diets exhibit distinct metabolic responses, as evidenced by the separation of various physiological parameters. Despite that all groups are significantly separated from each other, the groups TRP_Ø and CTRL_stress are in close proximity to one another, sharing a similar physiological profile in which hepatic lipid content decreased (present results) while cortisol levels enhanced (Machado et al. 2022). Interestingly, when stressed fish were provided a diet supplemented with tryptophan, their plasma cortisol profile and their energy reserves were in opposite trends.

PK, a key enzyme in the glycolytic pathway, plays a vital role by catalyzing the ultimate step of glycolysis, mediating the conversion of phosphoenolpyruvate into pyruvate. This enzymatic reaction yields adenosine triphosphate, thereby facilitating energy production within the cells (Alonso et al. 1985). Jerez-Cepa et al. (2019) demonstrated that administration of the glucocorticoid dexamethasone increased hepatic pyruvate demands in *Sparus aurata* juveniles by stimulating the activities of PK and fructose bi-phosphatase (FBP). These enzymatic changes are directly associated to glycolysis and gluconeogenesis enhancement, which display a pivotal function in maintaining stable energy levels (Faught et al. 2016; Jerez-Cepa et al. 2019). In our results, stress (space-confinement conditions) clearly demonstrates the need for increasing the hepatic pyruvate demands in fish fed CTRL and unstressed fish fed tryptophan (positively loaded by PK on the discriminant analysis). However, tryptophan supplementation in stressed fish was able to counteract the need for more PK, as expected for a control group (CTRL_ Ø) at homeostatic conditions.

Besides cortisol, glucose levels are known to increase after an acute stress as a classical metabolic biomarker as mentioned by Wendelaar Bonga (1997). Cortisol effects on carbohydrate metabolism reflect on increasing glucose by glycogenolysis or gluconeogenesis (Vijayan et al. 2010). In line with this, our results demonstrate that fish kept under crowding conditions and fed tryptophan exhibited lower hepatic glucose levels at 72 h post-injection as well as decreased plasma cortisol (parallel approach Machado et al. 2022), thereby supporting the hypothesis of the modulatory role of tryptophan to a homeostatic condition. Likewise, it was reported a reduction in glucose and cortisol levels in salmon and Atlantic cod that were fed a tryptophan-supplemented diet for 7 days and subsequently subjected to crowding and confinement stress (Basic et al. 2013a; 2013b; Höglund et al. 2005).

As mentioned before, two different, recently published analytical approaches to the hereby presented experiment (Machado et al. 2022 and Peixoto et al. 2024) have emphasized the modulatory role of tryptophan on the HPI axis response and central serotonergic activity. Furthermore, they have highlighted the inhibitory effect of dietary tryptophan supplementation on cortisol production induced by bacterial injection. Integrating these findings with the results obtained in the present study, it can be hypothesized that the tryptophan-induced attenuation of metabolic demands in stressed fish could be attributed to the interplay between serotonergic activity and the HPI axis. However, further research will be necessary in order to probe this hypothesis.

## Conclusion

In conclusion, dietary supplementation with tryptophan seems, at least after a short-time feeding trial, to modulate metabolism impacting whole-body homeostasis in fish under stress conditions. Fish fed tryptophan-supplemented diets exhibited distinct metabolic profiles compared to those fed CTRL. Fish fed TRP and reared under control conditions exhibited elevated hepatic glucose (present results) and plasma cortisol levels (parallel approach Machado et al. 2022), indicating activation of the stress axis. When animal density was high, fish fed CTRL reduced gradually lipid oxidative enzyme activity (HOAD) following i.p. *Phdp* injection to basal levels, which seems to suggest that acute stress triggered a feedback mechanism in lipid catabolism after bacterial challenge in stressful conditions. On the other hand, stressed fish fed tryptophan decreased hepatic glucose (present results) and cortisol levels (parallel approach Machado et al. 2022, as well as unchanged HOAD activity patterns (present results), suggesting an inhibitory and modulatory role of tryptophan on the stress response. Overall, these findings suggest that tryptophan supplementation for a short period of time in the diets of stressed fish mildly attenuates their metabolic responses to an acute inflammation, potentially alleviating the negative effects of stress. Further research is needed to explore the underlying mechanisms and to optimize tryptophan supplementation strategies in terms of timing and via of administration for improved stress management in aquaculture practices.

## Supplementary Information

Below is the link to the electronic supplementary material.

Supplementary file1 (DOCX 37 KB)

## Data Availability

No datasets were generated or analysed during the current study.

## Abbreviations

CTRL: Control diet
TRP: CTRL-based diet supplemented with tryptophan
i.p.: Intraperitoneally injected
HOAD: 3-Hydroxiacil-CoA dehydrogenase (EC 1.1.1.35)
5-HT: Serotonin (5-hydroxytryptamine)
HPI: Hypothalamus–pituitary–interrenal
DA: Multivariate canonical discriminant analysis
*tph1α*: Tryptophan hydroxylase
*htr2a*: Serotonergic receptor
*pomca*: Pro-opiomelanocortin a-like
*gr1*: Glucocorticoid receptor 1
ACTH: Adrenocorticotropic hormone
*Phdp*: *Photobacterium damselae piscicida*
TAG: Triglycerides
HK: Hexokinase (EC 2.7.1.1)
PK: Pyruvate kinase (EC 2.7.1.40)
tGP and aGP: Glycogen phosphorylase total and active (EC 2.4.1.1)
LDH: Lactate dehydrogenase (EC 1.1.1.27)
SD: Standard deviation
