# Correction to: Tryptophan‑supplemented diet modulates the metabolic response of European seabass (*Dicentrarchus labrax*) juveniles reared under space‑confined conditions and submitted to acute inflammation

**DOI:** 10.1007/s10695-025-01451-9

**Published:** 2025-01-29

**Authors:** Diogo Peixoto, Juan Antonio Martos‑Sitcha, Benjamín Costas, Rita Azeredo, Juan Miguel Mancera

**Affiliations:** 1 CIIMAR - Centro Interdisciplinar de Investigação Marinha E Ambiental, Av. General Norton de Matos S/N, 4450‑208 Matosinhos, Portugal; 2 ICBAS - Instituto de Ciências Biomédicas Abel Salazar, Universidade Do Porto, Porto, Portugal; 3 Departamento de Biología, Facultad de Ciencias del Mar y Ambientales, Instituto Universitario de Investigación Marina (INMAR), CEIMAR-Universidad de Cádiz, Cádiz, Spain

**Correction to: Fish Physiol Biochem (2025) 51:1–14**

10.1007/s10695-024-01427-1

The original version of this article unfortunately contained mistakes introduced during the production process. The corrections are given in the following list:

- 1. The wrong figure appeared as Fig. 2;

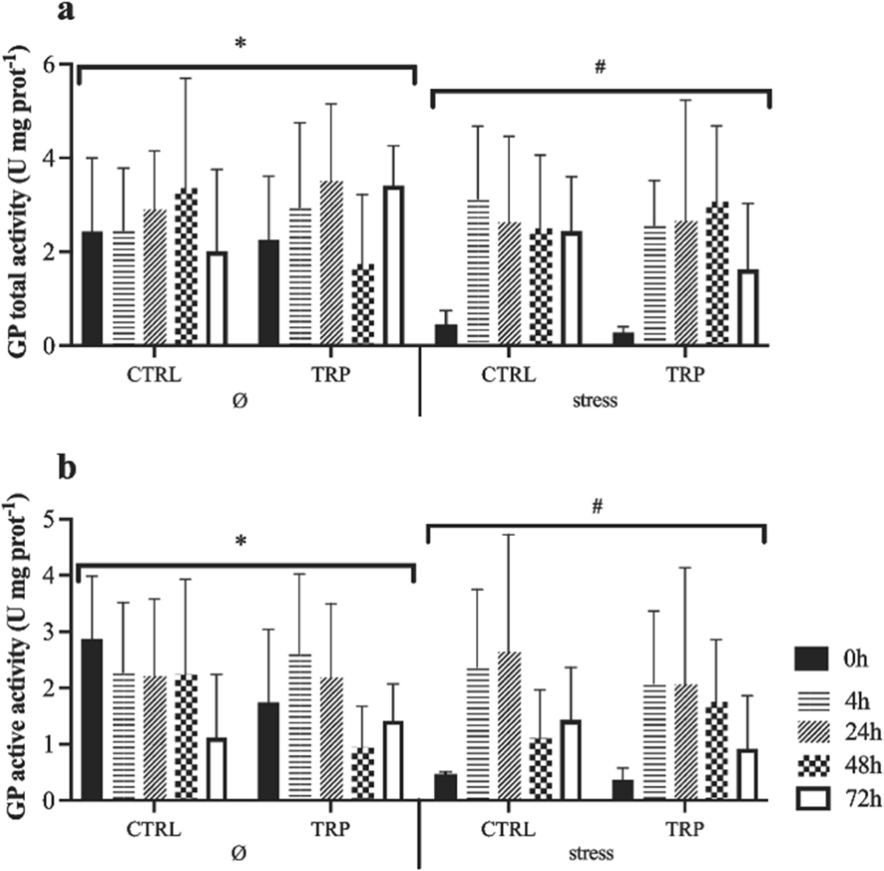


the figure should have appeared as shown below:
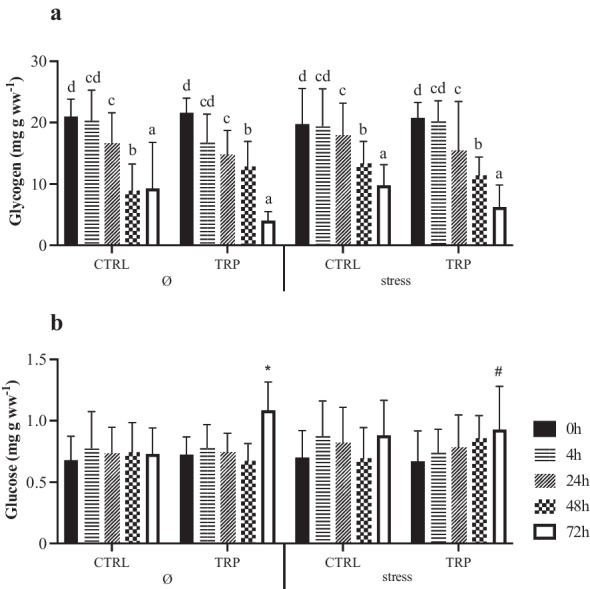

- 2. In the funding information, ‘funded by MCIN/AEI/10.13039/501100011033’ should have read ‘funded by MCIN/AEI/10.13039/501100011033’

The original article has been corrected.

