# Supplementary material for: Tryptophan-supplemented diet modulates the metabolic response of European seabass (Dicentrarchus labrax) juveniles reared under space-confined conditions and submitted to acute inflammation
Source: Fish Physiol Biochem. 2024 Dec 11;51(1):10. doi: 10.1007/s10695-024-01427-1 (PMC11634932; doi:10.1007/s10695-024-01427-1)
Supplement: Supplementary file 1 — Supplementary file1 (DOCX 37 KB) [file 10695_2024_1427_MOESM1_ESM.docx]

**Tryptophan-supplemented diet modulates the metabolic response of European seabass (*Dicentrarchus labrax*) juveniles reared under space-confined conditions and submitted to acute inflammation**

Diogo Peixoto^1,2,3^, Juan Martos-Sitcha^3^, Benjamín Costas^1,2*^, Rita Azeredo^1,2^, Juan Miguel Mancera^3^

^1^ CIIMAR - Centro Interdisciplinar de Investigação Marinha e Ambiental, Matosinhos, Portugal.

^2^ ICBAS - Instituto de Ciências Biomédicas Abel Salazar, Universidade do Porto, Porto, Portugal.

^3^ Departamento de Biología, Facultad de Ciencias del Mar y Ambientales, Instituto Universitario de Investigación Marina (INMAR), CEIMAR-Universidad de Cádiz, Cádiz, Spain

^*^Corresponding authors - Benjamín Costas | E-mail: [bcostas@ciimar.up.pt](mailto:bcostas@ciimar.up.pt)

Address: Av. General Norton de Matos s/n 4450-208 Matosinhos, Portugal

**Supplementary File**

**Table S1.** Liver metabolites of European seabass fed experimental diets under stressful conditions or not (Ø) or 15 days, and subsequently i.p. injected with *Phpd*. Fish were sampled before (0 hours post-injection) and after (4, 24, 48 and 72 hours post-injection) the i.p. *Phpd* injection.

|  | **Ø** | | | | | | | | | | |
| --- | --- | --- | --- | --- | --- | --- | --- | --- | --- | --- | --- |
|  | **CTRL** | | | | |  | **TRP** | | | | |
|  | **0h** | **4h** | **24h** | **48h** | **72h** |  | **0h** | **4h** | **24h** | **48h** | **72h** |
| Lactate (mg g ww^-1^) | 0.68 ± 0.2 | 0.78 ± 0.3 | 0.74 ± 0.21 | 0.74 ± 0.24 | 0.73 ± 0.21 |  | 0.73 ± 0.14 | 0.78 ± 0.19 | 0.74 ± 0.15 | 0.67 ± 0.14 | 1.09 ± 0.23 |
| Triglycerides (mg g ww^-1^) | 71.44 ± 12.64 | 99.32 ± 10.86 | 92.01 ± 24.63 | 89.4 ± 22.44 | 100.06 ± 30.22 |  | 74.69 ± 18.76 | 95.45 ± 19.08 | 85.38 ± 24.83 | 89.53 ± 15.76 | 91.01 ± 29.72 |
| Glycogen (mg g ww^-1^) | 20.98 ± 2.85 | 20.42 ± 4.85 | 16.56 ± 5.03 | 8.92 ± 4.33 | 9.29 ± 7.5 |  | 21.58 ± 2.41 | 16.83 ± 4.53 | 14.74 ± 3.99 | 12.83 ± 4.09 | 4.03 ± 1.48 |
| Glucose (mg g ww^-1^) | 0.68 ± 2 | 0.78 ± 0.3 | 0.74 ± 0.21 | 0.74 ± 0.24 | 0.73 ± 0.21 |  | 0.73 ± 0.14 | 0.78 ± 0.19 | 0.75 ± 0.15 | 0.67 ± 0.14 | 1.09 ± 0.23* |

|  | **Stress** | | | | | | | | | | |
| --- | --- | --- | --- | --- | --- | --- | --- | --- | --- | --- | --- |
|  | **CTRL** | | | | |  | **TRP** | | | | |
|  | **0h** | **4h** | **24h** | **48h** | **72h** |  | **0h** | **4h** | **24h** | **48h** | **72h** |
| Lactate (mg g ww^-1^) | 0.69 ± 0.22 | 0.88 ± 0.28 | 0.82 ± 0.29 | 0.69 ± 0.25 | 0.88 ± 0.28 |  | 0.67 ± 0.25 | 0.74 ± 0.19 | 0.78 ± 0.26 | 0.86 ± 0.19 | 0.93 ± 0.35 |
| Triglycerides (mg g ww^-1^) | 65.39 ± 11.95 | 86.54 ± 19.69 | 103.62 ± 29.77 | 77.33 ± 14.46 | 84.22 ± 23.75 |  | 71.13 ± 11.21 | 96.29 ± 21.21 | 102.5 ± 24.04 | 91.56 ± 18.27 | 79.59 ± 25.83 |
| Glycogen (mg g ww^-1^) | 19.74 ± 5.81 | 19.53 ± 6 | 17.88 ± 5.3 | 13.32 ± 3.59 | 9.77 ± 3.37 |  | 20.79 ± 2.47 | 20.29 ± 3.29 | 15.4 ± 8.06 | 11.37 ± 3.03 | 6.23 ± 3.6 |
| Glucose (mg g ww^-1^) | 0.7 ± 0.22 | 0.88 ± 0.28 | 0.82 ± 0.29 | 0.69 ± 0.25 | 0.88 ± 0.28 |  | 0.67 ± 0.24 | 0.74 ± 0.19 | 0.78 ± 0.26 | 0.86 ± 0.19 | 0.93 ± 0.35# |

| **Multifactorial ANOVA** |  |  | Sampling Time |  | Diet x Sampling Time | Stress x Sampling Time | Diet x Stress x Sampling Time | Sampling time | | | | | Stress | |
| --- | --- | --- | --- | --- | --- | --- | --- | --- | --- | --- | --- | --- | --- | --- |
|  | Diet | Stress |  | Diet x Stress |  |  |  |  |  |  |  |  |  |  |
|  |  |  |  |  |  |  |  | 0h | 4h | 24h | 48h | 72h | Ø | Stress |
| Lactate (mg g ww^-1^) | ns | ns | ns | ns | ns | ns | ns |  |  |  |  |  |  |  |
| Triglycerides (mg g ww^-1^) | ns | ns | <0.001 | ns | ns | ns | ns | a | b | b | b | b |  |  |
| Glycogen (mg g ww^-1^) | ns | ns | <0.001 | ns | ns | ns | ns | d | cd | c | b | a |  |  |
| Glucose (mg g ww^-1^) | ns | <0.001 | <0.05 | ns | ns | ns | <0.05 | a | b | ab | ab | ab | * | # |

Values are presented as means ± SD (n=8). *P*-values from three-way ANOVA (*p* ≤ 0.05). If the interaction was significant, Tukey *post-hoc* test was used to identify differences among treatments. Low case letters indicate differences attributed to sampling time. Different symbols denote significant differences between rearing conditions.

**Table S2.** Liver metabolic-related enzymes of European seabass fed experimental diets under stressful conditions or not (Ø) or 15 days, and subsequently i.p. injected with *Phpd*. Fish were sampled before (0 hours post-injection) and after (4, 24, 48 and 72 hours post-injection) the i.p. *Phpd* injection. HOAD – 3-hidorxiacil-CoA dehydrogenase (EC 1.1.1.35) activity, HK – hexokinase (EC 2.7.1.1) activity, PK – pyruvate kinase (EC 2.7.1.40) activity, tGP – total glycogen phosphorylase (EC 2.4.1.1) and aGP – active glycogen phosphorylase (EC 2.4.1.1).

|  | **Ø** | | | | | | | | | | |
| --- | --- | --- | --- | --- | --- | --- | --- | --- | --- | --- | --- |
|  | **CTRL** | | | | |  | **TRP** | | | | |
|  | **0h** | **4h** | **24h** | **48h** | **72h** |  | **0h** | **4h** | **24h** | **48h** | **72h** |
| HOAD (U mg protein^-1^) | 1.44 ± 0.35 | 1.87 ± 0.82 | 1.32 ± 0.69 | 1.46 ± 0.9 | 1.99 ± 1.07 |  | 1.69 ± 0.59 | 1.73 ± 0.61 | 1.4 ± 0.72 | 1.35 ± 0.89 | 1.34 ± 0.28 |
| HK (um mg protein^-1^) | 112.44 ± 35.78 | 112.77 ± 22.57 | 137.2 ± 51.3 | 101.78 ± 52.94 | 131.53 ± 41.65 |  | 111.86 ± 44.66 | 94.9 ± 43.44 | 141.53 ± 50.91 | 98.53 ± 41.9 | 85.03 ± 58.43 |
| PK (%) | 80.83 ± 10.16 | 75.13 ± 2.1 | 79.32 ± 12.19 | 64.76 ± 9.12 | 70.86 ± 13.01 |  | 80.56 ± 6.7 | 85.18 ± 8.26 | 80.21 ± 16.04 | 70.9 ± 19.18 | 79.07 ± 12.06 |
| tGP (U mg protein^-1^) | 2.43 ± 1.57 | 2.46 ± 1.32 | 2.89 ± 1.25 | 3.36 ± 2.35 | 2.01 ± 1.74 |  | 2.25 ± 1.37 | 2.95 ± 1.8 | 3.51 ± 1.64 | 1.74 ± 1.48 | 3.41 ± .85 |
| aGP (U mg protein^-1^) | 2.87 ± 1.11 | 2.28 ± 1.24 | 2.2 ± 1.38 | 2.24 ± 1.68 | 1.12 ± 1.12 |  | 1.74 ± 1.3 | 2.62 ± 1.41 | 2.18 ± 1.31 | 0.93 ± 0.74 | 1.12 ± 1.12 |

|  | **Stress** | | | | | | | | | | |
| --- | --- | --- | --- | --- | --- | --- | --- | --- | --- | --- | --- |
|  | **CTRL** | | | | |  | **TRP** | | | | |
|  | **0h** | **4h** | **24h** | **48h** | **72h** |  | **0h** | **4h** | **24h** | **48h** | **72h** |
| HOAD (U mg protein^-1^) | 2.85 ± 1.56Bb | 1.8 ± 0.96ab | 1.91 ± 0.79ab | 1.32 ± 0.9a | 0.89 ± 0.31Aa |  | 1.52 ± 0.41A | 2.2 ± 1.02 | 2.08 ± 1.15 | 1.26 ± 0.89 | 1.95 ± 0.96B |
| HK (um mg protein^-1^) | 138.62 ± 44.41 | 142.21 ± 39.18 | 115.92 ± 64.13 | 113.37 ± 59.46 | 83.66 ± 31.92 |  | 148.7 ± 42.89 | 120.99 ± 39.3 | 151.32 ± 39.38 | 125.67 ± 56.96 | 122.86 ± 27.24 |
| PK (%) | 74.88 ± 13.98 | 86.2 ± 6.33 | 83.43 ± 13.2 | 73.41 ± 4.83 | 82.83 ± 8.23 |  | 82.83 ± 8.23 | 85.32 ± 4.24 | 84.13 ± 10.39 | 76.23 ± 18.06 | 81.15 ± 14.83 |
| tGP (U mg protein-1) | 0.45 ± 0.3 | 3.13 ± 1.54 | 2.62 ± 1.84 | 2.49 ± 1.57 | 2.44 ± 1.16 |  | 0.28 ± 0.13 | 2.57 ± 0.95 | 2.65 ± 2.58 | 3.06 ± 1.62 | 1.62 ± 1.41 |
| aGP (U mg protein^-1^) | 0.46 ± 0.04 | 2.37 ± 1.38 | 2.63 ± 1.09 | 1.11 ± 0.85 | 1.42 ± 0.94 |  | 0.37 ± 0.21 | 2.08 ± 1.29 | 2.06 ± 2.08 | 1.75 ± 1.11 | 0.92 ± 0.94 |

**Table S2.** Continued.

| **Multifactorial ANOVA** |  |  | Sampling Time |  | Diet x Sampling Time | Stress x Sampling Time | Diet x Stress x Sampling Time | Sampling time | | | | | Stress | | Diet x Stress | | | |
| --- | --- | --- | --- | --- | --- | --- | --- | --- | --- | --- | --- | --- | --- | --- | --- | --- | --- | --- |
|  | Diet | Stress |  | Diet x Stress |  |  |  |  |  |  |  |  |  |  | CTRL | | TRP | |
|  |  |  |  |  |  |  |  | 0h | 4h | 24h | 48h | 72h | Ø | Stress | Ø | Stress | Ø | Stress |
| HOAD (U mg protein^-1^) | ns | ns | <0.05 | ns | ns | ns | <0.05 | ab | b | ab | a | ab |  |  |  |  |  |  |
| HK (um mg protein^-1^) | ns | <0.05 | <0.05 | <0.05 | ns | ns | ns | ab | ab | b | ab | ab | # | * |  |  | # | * |
| PK (%) | ns | <0.05 | <0.05 | ns | ns | ns | ns | ab | b | b | a | ab | # | * |  |  |  |  |
| tGP (U mg protein^-1^) | ns | <0.05 | ns | ns | ns | ns | ns |  |  |  |  |  | * | # |  |  |  |  |
| aGP (U mg protein^-1^) | ns | <0.05 | ns | ns | ns | ns | ns |  |  |  |  |  | * | # |  |  |  |  |

Values are presented as means ± SD (n=8). *P*-values from three-way ANOVA (*p* ≤ 0.05). If the interaction was significant, Tukey *post-hoc* test was used to identify differences among treatments. Different capital letters stand for significant differences attributed to dietary treatment. Low case letters indicate differences attributed to sampling time. Different symbols denote significant differences between rearing conditions.

**Table S3.** Canonical discriminant analysis of European seabass metabolic response after 15 days of feeding and after 4, 24, 48 and 72 hours after i.p. *Phpd* injection.

**Table S3.1** – Mahalanobis distances of each group.

|  | CTRL_ Stress | CTRL_ Ø | TRP_ Stress | TRP_ Ø |
| --- | --- | --- | --- | --- |
| CTRL_ Stress | 0 | 1.849 | 1.543 | 1.053 |
| CTRL_ Ø | 1.849 | 0 | 3.187 | 1.162 |
| TRP_ Stress | 1.543 | 3.187 | 0 | 3.335 |
| TRP_ Ø | 1.053 | 1.162 | 3.335 | 0 |

**Table S3.2** – *p*-value for Fisher distances.

|  | CTRL_ Stress | CTRL_ Ø | TRP_ Stress | TRP_ Ø |
| --- | --- | --- | --- | --- |
| CTRL_ Stress | 1 | <0.0001 | 0.000 | 0.011 |
| CTRL_ Ø | <0.0001 | 1 | <0.0001 | 0.003 |
| TRP_ Stress | 0.000 | <0.0001 | 1 | <0.0001 |
| TRP_ Ø | 0.011 | 0.003 | <0.0001 | 1 |

**Table S3.3** – Variables correlation/factors.

|  |  | F1 | F2 | F3 |
| --- | --- | --- | --- | --- |
| Hepatic metabolites | TAG | -0.075 | -0.330 | 0.503 |
|  | Lactate | 0.110 | 0.211 | 0.304 |
|  | Glucose | -0.440 | 0.043 | 0.737 |
|  | Glycogen | -0.020 | -0.238 | -0.279 |
| Hepatic related-enzymes | HOAD | 0.164 | -0.038 | -0.254 |
|  | HK | 0.204 | -0.488 | -0.095 |
|  | PK | 0.382 | 0.232 | -0.015 |
|  | tGP | -0.222 | -0.041 | 0.116 |
|  | aGP | -0.338 | -0.352 | -0.053 |
| Plasma | Cortisol | -0.787 | 0.248 | -0.304 |
